# Editorial: International day of happiness: happiness and wellbeing in the age of climate catastrophe

**DOI:** 10.3389/fpsyg.2024.1497347

**Published:** 2024-10-08

**Authors:** Andrew H. Kemp, Zoe Fisher, Panu Pihkala

**Affiliations:** ^1^School of Psychology, Faculty of Medicine, Health and Life Science, Swansea University, Swansea, United Kingdom; ^2^Community Brain Injury Service, Morriston Hospital, Swansea, United Kingdom; ^3^Climate Action Research Institute, Swansea University, Swansea, United Kingdom; ^4^Faculty of Theology and HELSUS Sustainability Science Institute, University of Helsinki, Helsinki, Finland

**Keywords:** climate psychology, holistic wellbeing, social ecological models, climate-related emotions, climate action

The quest for happiness and wellbeing amid global environmental crises presents complex challenges, but also opportunities. Initiated on the International Day of Happiness in 2022, our Research Topic invited reflection on various aspects related to this quest. Related themes are discussed in the “Climate Change and Happiness” podcast (see: https://climatechangeandhappiness.com/), one of the inspirations for this Research Topic.

What will happen to happiness amidst the intensifying climate crisis? The future looks daunting: threats to happiness include forced climate displacement of entire populations, potentially exceeding 140 million people by 2050 (e.g., Xu et al., 2020), predictions of the first mass biodiversity extinction event caused entirely by humans (e.g., Cowie et al., 2022), the intensification of climate-related conflicts and wars (e.g. Mach et al., 2019; Li et al., 2021) and the collapse of agricultural systems leading to existential risks for humanity (e.g., Richards et al., 2021). These predictions are already materializing through migration crises, species loss, international tensions and food shortages. The climate crisis is here, and some argue that it should already be called a climate catastrophe (e.g. Boyd, 2023).

**Figure 1:**
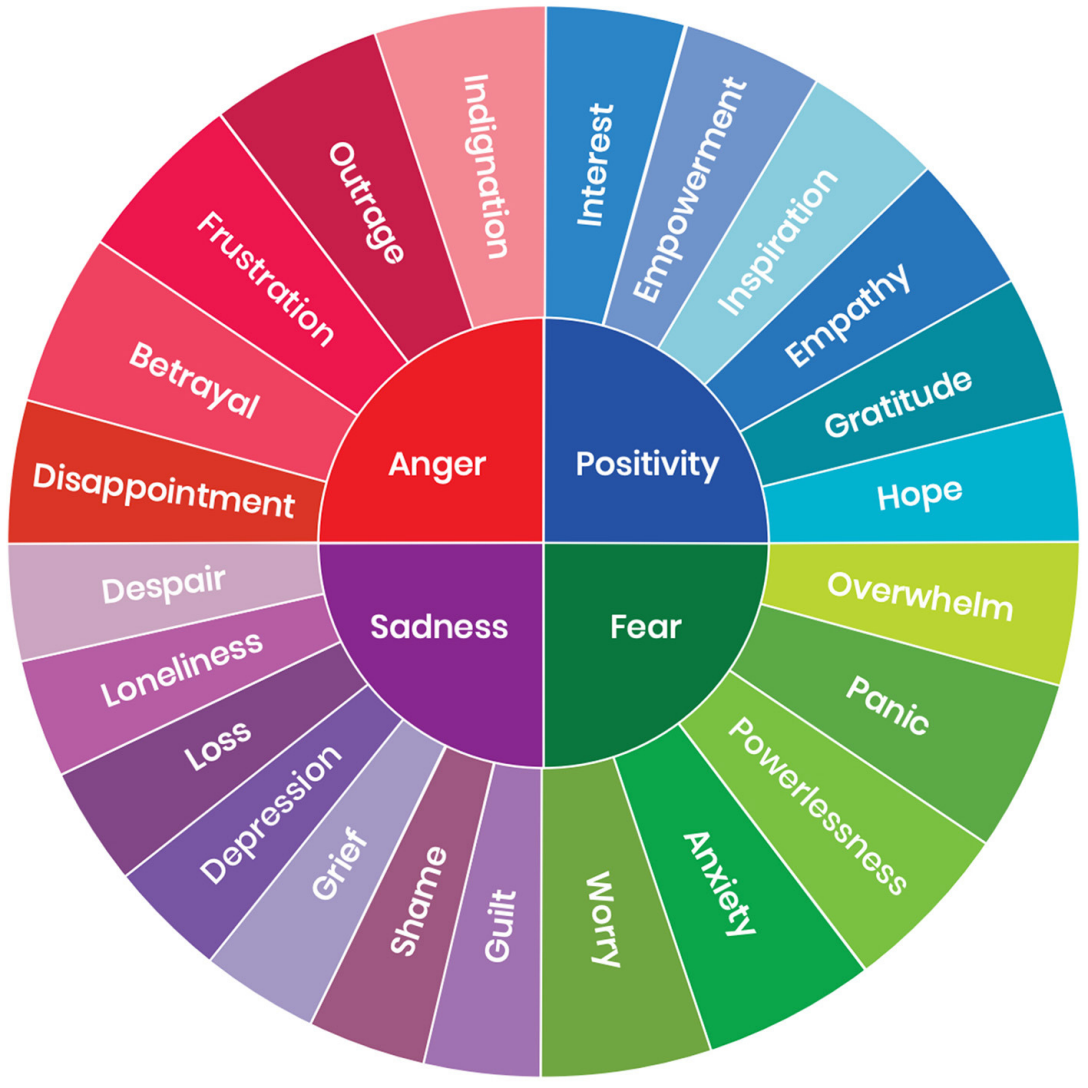
The Climate Emotions Wheel by Panu Pikhala, licensed under CC BY-NC-SA. Reprinted from https://www.climatementalhealth.net/wheel.

The situation evokes many kinds of “climate-related emotion,” including aspects of anger, sadness, fear and even positive feelings including gratitude (for example, about those who participate in climate action), empowerment and hope (Figure 1). Such emotions can impact on our capacity to function and are associated with pro-environmental behaviors and wellbeing in complex ways (Pihkala, 2022; Brosch and Sauter, 2023).

Climate-related emotions can adversely impact on wellbeing including anxiety, fear, worry, grief, sadness, and guilt or shame (Kurth and Pihkala; Pihkala, 2024). Yet, these emotions also have the potential to drive meaningful action at multiple levels of scale. For instance, emotionally evocative letters of apology to future generations (see https://youtu.be/yJvczRkQHK0?si=HkN7humvYND3vZt6), reflect an expression of increasing public shame in climate change professionals—as well as frustration and catharsis—that may ultimately foster deeper engagement within individuals, communities and institutions in addressing the complex barriers that have delayed climate action.

**Figure 2:**
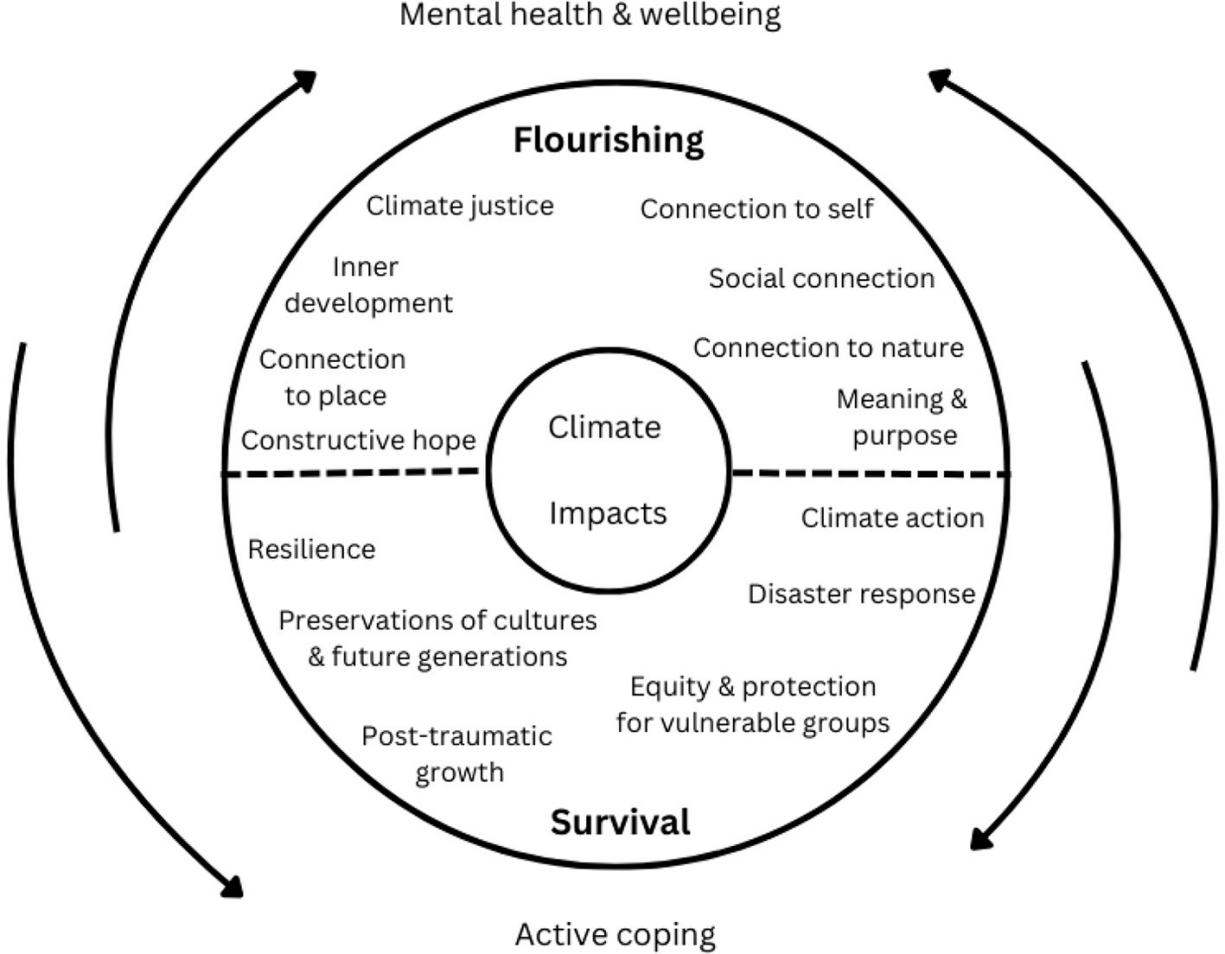
The interconnections between climate change, survival and wellbeing. Adapted from Doherty (2018) and used with permission.

Climate change has been described as a “super-wicked problem” (Cross and Congreve, 2021), a threat multiplier that will inevitably constrain our capacity for wellbeing (Lawrance et al., 2022). Wellbeing is an equally complex construct (Wilkie et al., 2022), encompassing positive emotions (Fredrickson, 2004), sense of meaning (Wong, 2016), inner development (Woiwode et al., 2021), social identity (Jetten et al., 2017), social cohesion (Delhey and Dragolov, 2016), nature connection (Martin et al., 2020), and pro-environmental behaviors (Capstick et al., 2022). This work has prompted researchers to reflect deeply on how research on wellbeing—broadly defined—can support societies to address impending climate-related disasters. It has facilitated the integration of historically distinct sub-disciplines, including positive psychology, acceptance and commitment therapy, and climate psychology (Kemp and Edwards, 2022). Figure 2 is illustrative of some considerations relating to the interconnections between climate change, survival and wellbeing. This is not only a meaningful intellectual enterprise, but one with significant implications for societal transformation (Kemp and Fisher, 2022).

Below, we briefly comment on the seven published scholarly articles in Research Topic, which provide insights into addressing climate change through the lens of wellbeing.

We begin with the contribution from Isham, Morgan et al. who advocate for a wellbeing-focused approach to climate psychology, underpinned by a social ecological understanding of distress and wellbeing that places the individual within the context of their groups and systems. The Power Threat Meaning Framework (PTMF) (Johnstone and Boyle, 2018) provides a non-pathological framing of distress and highlights how individuals and their communities can harness their strengths and resources for effective responses to threats, creating opportunities for wellbeing despite considerable distress. Complementing this approach is the holistic model of wellbeing, the GENIAL framework (Kemp and Fisher, 2022) which emphasizes a key role for connections to self, others, and nature (see also Bristow et al., 2022; Woiwode et al., 2021), reflecting the complexity of wellbeing across individual, collective, and planetary levels. Initiatives such as mindfulness training, prosocial practices, and nature-based activities present opportunities to support these various aspects of wellbeing. This work reinforces the necessity of individual-focused interventions alongside community-level initiatives focused on the unique impacts on climate change at local levels and related opportunities for positive change through a systems-informed lens (see also Kern et al., 2020; Lomas et al., 2020, 2024).

A key idea here is how climate-related emotions can drive such change. In this regard, Kurth and Pihkala's paper highlights how eco-anxiety can trigger ecological consciousness and motivate environmental stewardship and agency. They problematize the construct of eco-anxiety, which previous authors have used to describe a variety of emotions including anxiety, fear, guilt, and sadness, each influencing behaviors in different ways. Eco-anxiety can lead to various responses, including approach and avoidance. They identify ‘practical eco-anxiety' as an approach-related component of eco-anxiety that may serve to promote pro-environmental attitudes and actions supporting a move toward a positive sustainable future. The paper emphasizes reappraisal strategies, mindfulness training, and connecting with others as ways to harness practical eco-anxiety.

Similarly, Isham, Elf et al.'s discusses how self-transcendent experiences (STEs) might promote “ecological wellbeing,” defined as the wellbeing of the planet and its inhabitants. The authors note that modern lifestyles contribute to ecological deterioration through unsustainable consumption and a disconnection from nature. Despite past efforts, necessary carbon reductions have not been achieved. STEs, including flow states, awe, mindfulness, and even psychedelic experiences, provide pathways to ecological wellbeing. However, the authors emphasize concerns over utilizing STEs for commercial aims (e.g., McMindfulness'), which may instead reinforce materialism, overconsumption and disconnection from nature.

The article by Steentjes and Roberts focuses on the mental health of climate change professionals, a group that plays a crucial role in navigating the crisis. The authors highlight how guilt and perceived inefficacy threaten the wellbeing of these individuals to such an extent that many professionals feel unsupported and are considering leaving the sector. The authors call for systemic changes to prevent burnout and promote resilience (Steentjes and Roberts), including the creation of safe spaces for creative community engagement and the exploration of meaningful action in response to the climate crisis, transforming anxiety and despair into motivational drivers for action.

Moving beyond individual experiences and emotions, Bellehumeur and Carignan introduce relational environmental metaphors to enhance public engagement, using familiar comparisons to deepen emotional connections and understanding of environmental issues. They critique “alarmist” climate metaphors (i.e., fear-based messaging), which may reduce collaborative engagement and negatively impact mental health, instead advocating for relational metaphors (hope-based messaging) that elicit positive eco-emotions like connection and compassion. This paper offers a valuable perspective on how language can serve as either a barrier or an enabler, influencing whether people move toward or away from desirable changes. While we acknowledge that we began with what might be described as “fear-based messaging” in this paper, it is important to differentiate between scholarly discussion and the communication of research findings to the wider public. We further suggest that the increasingly pessimistic outcomes reported in climate science reinforce an urgent need to better integrate climate action with wellbeing research agendas.

In keeping with a focus on community-based solutions, Marks et al. report on a school-based workshop using collective storytelling to build community resilience and hope. They argue that the desire to protect young people by not discussing climate concerns or delivering climate change education can leave them feeling unsupported, marginalized and disenfranchised (see also Atkinson and Ray, 2024). Their workshop, framed within narrative identity theory and Youth Participatory Action Research, revealed a mixture of painful and positive emotions, emphasizing the importance of eco-empathy and eco-compassion in fostering prosocial action and care.

Finally, Weijers and Agar focus on multiple domains of scale, highlighting the need for essential behavioral and policy reforms. They critique the over-reliance on climate technology (Weijers and Agar) and warn against “horizon bias,” which could delay necessary climate action. The authors advocate for a balanced approach that combines technological solutions with substantial lifestyle and policy changes. They caution that over-reliance on science and technology may foster complacency, thereby undermining efforts to reduce carbon emissions. Instead, they emphasize the need for immediate, large-scale emissions reduction efforts through climate-friendly actions and collective engagement.

Together, these perspectives reinforce the need for a more holistic approach to wellbeing that recognizes the interconnectedness of individual, collective, and planetary wellbeing. The published studies in this Research Topic showcase opportunities to align wellbeing at multiple levels of scale with the climate crisis, focused on empowering individuals and communities to harness their strengths, foster resilience and promote a shared sense of meaning and purpose. By reframing experiences through collective storytelling, and promoting a socially engaged mindfulness alongside community-driven initiatives, these studies suggest intriguing opportunities for positive change that will—we hope—promote the holistic wellbeing of self, others and nature.
